# Study protocol for a hybrid type 1 effectiveness-implementation trial testing virtual tobacco treatment in oncology practices [Smokefree Support Study 2.0]

**DOI:** 10.1186/s12889-022-13631-w

**Published:** 2022-07-15

**Authors:** Brett M. Goshe, Autumn W. Rasmussen, Lynne I. Wagner, JoRean D. Sicks, Ilana F. Gareen, Ruth C. Carlos, Benjamin A. Herman, Angela Wangari Walter, Susan Regan, Douglas E. Levy, Irene Mahon, Alona Muzikansky, Jordan M. Neil, Michelle Lui, Deepika Dilip, Laura Malloy, Irina Gonzalez, Lucy Finkelstein-Fox, Caitlin McCann, Elissa Perez, Jamie S. Ostroff, Elyse R. Park

**Affiliations:** 1 Department of Psychiatry, Massachusetts General Hospital / Harvard Medical School, 100 Cambridge Street, Suite 1600, Boston, MA 02114 USA; 2 Mongan Institute Health Policy Research Center, Massachusetts General Hospital, Boston, MA USA; 3 Health Promotion and Resiliency Intervention Research Program, Massachusetts General Hospital, Boston, MA USA; 4 Harvard Medical School, Boston, MA USA; 5 Wake Forest University School of Medicine, Winston-Salem, NC USA; 6 ECOG-ACRIN Biostatistics Center, Brown University, Providence, RI USA; 7 Department of Epidemiology and the Center for Statistical Sciences, Brown University School of Public Health, Providence, RI USA; 8 Michigan Medicine, University of Michigan, Ann Arbor, MI USA; 9 Department of Public Health, Zuckerberg College of Health Sciences, University of Massachusetts Lowell, Lowell, MA USA; 10 ECOG-ACRIN Cancer Research Group, American College of Radiology, Philadelphia, PA USA; 11 Biostatistics Center, Massachusetts General Hospital, Boston, MA USA; 12 Department of Family and Preventative Medicine, University of Oklahoma Health Sciences Center, Oklahoma City, OK USA; 13 Department of Psychiatry & Behavioral Sciences, Memorial Sloan Kettering Cancer Center, New York, NY USA

**Keywords:** Smoking Cessation, Tobacco Treatment, Implementing Tobacco Treatment, Cancer Care, Randomized Controlled Trial, Motivational Interviewing, Pharmacotherapy, NCORP

## Abstract

**Background:**

Persistent smoking among patients diagnosed with cancer is associated with adverse clinical outcomes, yet an evidence-based tobacco use intervention has not been well-integrated into cancer care in community oncology settings. This paper describes the protocol of a nation-wide clinical trial conducted by the ECOG-ACRIN National Cancer Institute (NCI) Community Oncology Research Program (NCORP) Research Base to assess the effectiveness of a virtual tobacco treatment intervention and the process of implementing tobacco treatment in NCORP community oncology settings.

**Methods/design:**

This two-arm, multisite (n: 49 NCORP sites) hybrid type 1 effectiveness-implementation randomized controlled trial compares the effectiveness of a Virtual Intervention Treatment (VIT) versus an Enhanced Usual Control (EUC) among English and Spanish speaking patients recently diagnosed with cancer, reporting current smoking and receiving care at a participating NCORP Community or Minority/Underserved Site. The VIT includes up to 11 virtual counseling sessions with a tobacco treatment specialist and up to 12 weeks of nicotine replacement therapy (NRT). The EUC arm receives a referral to the NCI Quitline. The primary study outcome is biochemically confirmed 7-day point prevalence smoking abstinence. Moderators of treatment effect will be assessed. The study evaluates implementation processes from participating NCORP site staff via survey, administrative, and focus group data, including reach, acceptability, appropriateness, fidelity, feasibility, adoption, cost and sustainability outcomes.

**Discussion:**

This trial will generate findings about the effectiveness of an evidence-based virtual tobacco treatment intervention targeting patients diagnosed with cancer and illuminate barriers and facilitators that influence implementing tobacco treatment into community oncology settings nationally. In the era of COVID-19, virtual care solutions are vital for maximizing access and utilization of tobacco treatment delivery.

**Trial registration:**

ClinicalTrials.gov (NCT03808818) on January 18th, 2019; Last update posted: May 21st, 2020.

## Introduction

### Background and rationale

Approximately 10 to 30% of adults report tobacco use at the time of their cancer diagnosis, and the majority who smoke continue to do so following diagnosis [1–4]. Smoking among individuals with a cancer diagnosis is associated with increased treatment toxicity, diminished effectiveness of cancer treatment, increased risk of recurrence and diagnosis of second primary cancer [5–12].Additionally, persistent smoking for those with a cancer diagnosis can cause increased risk of complications from surgery, radiation, and chemotherapy [13–19] and contribute to poor quality of life and decreased survival rates [1, 20, 21]. As such, promoting smoking cessation among individuals with a cancer diagnosis is a critical aspect of high quality cancer care [1, 22–25].

The National Comprehensive Cancer Network (NCCN) published smoking cessation guidelines [26] to promote cessation advice, counseling, and pharmacotherapy for cancer patients who report current smoking. However, only 30 to 40% of oncologists report assisting patients with quitting smoking [27–29]. There is a need for tobacco treatment delivery to be improved for patients receiving treatment in oncology settings.

Data from our preliminary trial (Smokefree Support Study 1.0) [30] demonstrated that an intensive tobacco treatment for newly diagnosed cancer patients is effective when delivered both in-person and remotely, via phone. Based on these findings, we proposed a trial that examines the efficacy of an intensive tobacco treatment which allows for face-to-face patient-counselor rapport (via videoconferencing delivery) while remaining accessible to those receiving their cancer care in rural, under-resourced, communities.

We describe the protocol for a randomized controlled trial (Smokefree Support Study 2.0) conducted in partnership with the National Cancer Institute (NCI) Community Oncology Research Program (NCORP) to assess the effectiveness of a virtual tobacco intervention and the process of implementing tobacco treatment in community oncology settings.

### Aims and objectives

The goal of this study is to test the effectiveness of a virtually delivered tobacco treatment intervention, in English and Spanish, in community cancer centers nationally. Aim 1 will assess treatment effectiveness by comparing 6-month biochemically confirmed 7-day point prevalence abstinence for participants randomly assigned to receive either the Virtual Intervention Treatment (VIT) or the Enhanced Usual Care (EUC). Aim 2 will assess the potential effect of moderators on treatment effectiveness. Aim 3 will assess the processes of implementing tobacco assessment and treatment interventions in community oncology sites.

### Study design

The Smokefree Support Study 2.0 is a two-arm randomized controlled trial utilizing a Hybrid Type 1 design [31] to test the effectiveness of the tobacco treatment interventions while also gathering information about the implementation process. Participants are randomly assigned in a 1:1 ratio to either the VIT (*n* = 154) or EUC (*n* = 154) intervention. Participants in VIT receive up to 11 counseling sessions with a tobacco treatment specialist (TTS) and can choose to receive up to 12 weeks of nicotine replacement therapy (NRT), patch and/or lozenge, over a 6 month treatment period. Participants in EUC receive a referral to the National Cancer Institutes (NCI) Smokers’ Quitline for free tobacco treatment counseling.

The Centralized Institutional Review Board (CIRB) of the National Cancer Institute (NCI) approved the study protocol, ECOG-ACRIN-EAQ171CD. Additional human subjects’ approvals were obtained by Massachusetts General Brigham (MGB), Memorial Sloan Kettering Cancer Center (MSKCC), and Brown University review boards. The trial was registered at ClinicalTrials.gov (NCT03808818).

### Conceptual frameworks

The VIT is grounded in two common theoretical frameworks: the Self-Regulation Model (SRM) [32], which is a model of coping with illness, and the Health Belief Model (HBM) [33], which is a model of how beliefs about personal health risks and resources impact behavior change. Proctor and colleagues’ (2011) [34] recommendations guided the measurement of implementation outcomes. Site staff surveys assess contextual factors influencing implementation and include organizational theory-informed and -validated measures [35, 36]. Site focus group interview guides are informed by the Consolidated Framework for Implementation Research (CFIR) [37] to evaluate barriers and facilitators for implementing and sustaining tobacco treatment in routine cancer care.

## Methods: participants, interventions, and outcomes

### Study setting

The ECOG-ACRIN Cancer Research Group (EA) is a scientific organization that designs and conducts cancer research. EA has a network of nearly 1,300 academic and community-based cancer centers and hospitals in the United States and internationally. The EA NCORP Research Base serves as a scientific hub for research conducted through the NCORP network, an expansive national network of cancer care sites dedicated to bringing clinical trials out of academic medical centers and into broader community oncology settings. NCORP is comprised of 32 Community Sites and 14 Minority/Underserved Sites, which collectively include over 900 sites and health care systems. This trial is open to any NCORP institution.

### Site recruitment

The study team hosted a series of informational webinars open to all NCORP sites and presented the study at biannual ECOG-ACRIN national meetings. Interested sites activated the study by 1) recording a brief outreach video of a site oncologist using a standardized script developed by the study team, 2) completing approximately 8–10 site staff baseline surveys (oncology clinicians and research staff) assessing key contextual factors such as their site’s commitment to offering tobacco treatment, and a brief description of their current tobacco treatment services, 3) completing training on data collection and transfer systems, and 4) identifying a Cancer Care Delivery Research (CCDR) lead who would attend monthly study conference call meetings. Forty-nine sub-affiliate sites across 17 NCORP sites activated the trial.

### Site staff eligibility

Staff eligibility is determined by the NCORP Principal Investigator and CCDR lead at each participating site and includes local clinical and administrative staff members who have knowledge of cancer care delivery and the challenges of providing tobacco treatment. Eligible staff participants are 1) English-speaking, 2) employed at the NCORP site for at least 3 months, and 3) able to provide informed consent to participate in this study.

### Patient eligibility

Patient eligibility criteria is intended to be broad and inclusive to maximize generalizability and reach. Patients are not required to want to quit at enrollment, but rather have a willingness to discuss their smoking with a tobacco treatment specialist (TTS). Eligibility criteria include adult (age 18 years or older) patients who 1) are diagnosed with any type of cancer within the past 4 months, 2) have smoked, even “a puff,” of a combustible cigarette in the last 30 days, 3) receive their oncology care at a participating NCORP site, 4) have access to the internet and camera-enabled device (e.g., smartphone/tablet/computer) for telehealth counseling sessions, and 5) are able to consent in English or Spanish. Additional exclusion criteria include ECOG performance status of 3 or above (measurement of patient’s level of functioning) or deemed medically unable to participate by the study investigators or patient’s oncology clinician.

### Patient recruitment

Eligible patients are identified and recruited by study staff at participating NCORP sites, according to each site’s determination of screening procedures for eligible patients. Some sites have automated systems for identifying current smokers whereas others conduct manual assessments. Identified patients’ charts are reviewed to verify they preliminarily meet the eligibility criteria, and eligibility is then confirmed via phone or in clinic. Study staff then share a brief recruitment video highlighting the importance of smoking cessation for patients diagnosed with cancer. Content for the recruitment video was developed and pilot-tested in a national sample of current smokers with a recent cancer diagnosis [38]. Different message frames (e.g., the risks of continued smoking and the benefits of participating in the trial) were examined to ensure the video was patient-centered and effective at promoting participation in the trial. Participants provide written informed consent, which outlines efforts to maintain confidentiality and limits of confidentiality (e.g., psychological emergency). Then they are able to complete a baseline survey in EA Systems for Easy Entry of Patient Reported Outcomes (EASEE-PRO), and are randomized 1:1 in blocks of four within NCORPs to a treatment arm. The EA automated system generates arm allocation and site staff enroll the participants. The system emails the arm assignment to the enrolling site staff. For participants randomized to the VIT arm, NCORP site staff securely transfers patient and provider information to MGB who informs the participants’ oncology care providers of the patients’ enrollment in the study. Participants randomized to the EUC arm receive a mailed letter with referral information to the NCI Quitline.

### Tobacco treatment interventions

#### Enhanced Usual Care (EUC)

Consistent with the current NCCN Smoking Cessation Guidelines [26], EUC patients receive a referral to the NCI Smokers’ Quitline for free tobacco treatment counseling. Participants referred to the Quitline receive a mailed letter by MGB staff with detailed instructions on how to access the Quitline services. The NCI Quitline provides the study team with a bi-annual report on participant engagement (e.g., dates and duration of calls) with Quitline services.

#### Virtual Intervention Treatment (VIT)

The VIT counseling protocol was adapted (e.g., updated with problem solving barriers to medication adherence; discussion of nicotine addiction and smoking-related stigma; and additional strategies for managing cravings) from the treatment manual used in the prior Smokefree Support Study 1.0 [30] and consists of 11 telehealth sessions (4 weekly; 4 biweekly; 3 monthly) that take approximately 30 min to complete. The sessions are conducted by TTS and guided by the well-established Motivational Interviewing (MI) strategy focusing on relevant tobacco treatment themes for patients newly diagnosed with cancer (e.g., helping to build and maintain quitting self-confidence; navigating sensitive topics like social support and stigma; and delivering information on the health benefits of quitting smoking) [39]. The sessions teach skills for managing cravings and mood, how to overcome barriers to NRT adherence, and provide participants encouragement for continued skills practice between sessions. Each counseling session is also formatted around the 5As counseling model (i.e., Ask, Advise, Assess, Assist, Arrange) [40]. Sessions conclude with reviewing the session content and participant goals, as well as reinforcing participant’s values-guided reasons for quitting/cutting back. The TTS documents session content and adherence in the counseling database.

#### Interventionist training and supervision

All counselors complete a 4-day Tobacco Treatment Specialist Training Program accredited by the Council for Tobacco Treatment Training Programs (CTTTP; https://ctttp.org/). The training covers a set of core competencies for tobacco treatment (e.g., assessment and treatment planning, counseling skills, and pharmacotherapy). Counselors are additionally trained on the study-specific VIT counseling protocol through a series of role plays (i.e., first as a participant of the program with a previously trained counselor and then in the role of the counselor). Weekly group supervisions are conducted to review all active cases. Additionally, counselors review a randomly selected, previously recorded counseling session and rate adherence to the treatment protocol and MI principles, using the treatment fidelity checklist [30, 41].

#### Initial VIT counseling session

During the initial session, the TTS provides a study overview, goals, and structure of the program; gathers the patient’s smoking history; assesses concerns about smoking; offers a personalized message to quit smoking; invites participants to rate the importance of quitting and their confidence in their ability to quit; discusses the pros and cons for quitting and continuing to smoke; and finally, evaluates participants’ readiness to quit. The TTS creates a tailored quit plan based on participants’ quit stage, classified into 3 categories: 1) Not ready to quit or make changes; 2) Not ready to quit, but ready to makes changes; and 3) Ready to quit.

#### Follow-up counseling sessions

Follow-up sessions are designed to build upon content presented in previous meetings. First, the TTS assesses the participant’s current level of stress and any updates to their cancer care. Stress management strategies are emphasized. At every session, the participant’s level of quit confidence and importance is assessed to help guide and monitor progress in making changes towards quitting. The TTS assesses use of cessation medications and addresses any barriers to medication use or adherence. Next, the TTS reviews progress on goals established in the previous session and introduces themes that can impact quitting goals (e.g., stress management, social support, and self-care). Table 1 provides more detail on each session’s content.

**Table 1:** Counseling protocol and content

Session #		Counseling Topics	Cessation Medication
1	Weekly	• Smoking assessment • Introduction to Stress management- stress coping • Barriers to quitting and strategies to enhance readiness • Nicotine and addiction • Medication education and assistance	• Introduction to NRT & use
2		• Cancer related care and distress, care team communication • Assess medication adherence and managing side effects • Knowledge about quitting at the time of diagnosis • Coping with cravings and withdrawal • Stress management- stress signs and coping	• NRT question/side effects
3		• Smoke free home and car • Social support • Stress management – mini relaxations	• Assess NRT use & 2^nd^ dose
4		• Introduce beginning with appreciations • Managing slips and relapses during/ following treatment • Stress management- belly breathing	• Review 2^nd^ dose/NRT fit
5	Bi-Weekly	• Smoking associated stigma and negative self-talk • Values clarification exercise • Stress management– single pointed focus exercise	• Assess adherence during treatment
6		• Resources for family/household members who smoke • Rewards and financial costs of smoking • Stress management- Mindful Awareness in daily life	• Assess adherence during/post treatment
7		• Risk of other forms of tobacco • Stress management- Mindfulness: Pause- Breathe-Reflect-Choose Exercise	• Assess adherence during/post treatment
8		• Pleasurable behaviors • Sleep and self-care	• Review NRT completion
9	Monthly	• Fear of recurrence • Managing physical symptoms	• Discuss if any continued NRT
10		• Managing cravings during/ following treatment • Picturing positive change	• Discuss if any continued NRT
11		• Stress and coping review • Managing slips and relapses review • Review overall smoking progress • Finalize smoking goals, relapse prevention • Post treatment Support	• Discuss if any continued NRT

#### Smoking cessation medication and advice

Participants assigned to the VIT can receive an initial 4-week supply of over-the-counter nicotine patches (7 mg, 14 mg or 21 mg) and/or mini-lozenges (2 mg or 4 mg) and up to two 4-week refills based on patient preferences at no cost. During the initial counseling session, the TTS provides advice and recommendations for use of NRT guided by a structured decision tree to review contraindications of the patch and lozenge. The use of cessation medication is encouraged but not required to continue participation in the study. Counselors also discuss other tobacco treatment medication available (e.g., varenicline, nicotine inhaler, nicotine gum) and encourage participants to speak with their healthcare provider if interested in these options. During the follow-up sessions, potential side effects of all cessation medications are monitored and addressed by the counselor.

### Outcomes

The primary outcome of this study is the clinical effectiveness of VIT relative to EUC, demonstrated by biochemically verified 7-day point-prevalence abstinence at 6 months. Secondary outcomes include self-reported 7-day smoking abstinence at 3 and 6 months and biochemically verified at 3-months. Exploratory outcomes include assessment of the implementation processes.

## Measures and data collection, sources, and timeline: clinical effectiveness outcome measures and data collection

### Biochemically verified abstinence (primary outcome) [42]

Seven-day point-prevalence smoking abstinence at 6-month (primary) and 3-month follow-up (secondary), is confirmed biochemically by salivary cotinine assays [30, 43, 44]. Saliva samples are requested from all participants who report 7-day point prevalence abstinence at 3-and/or 6-month follow-up and undergo sample processing. Patients receive a $40 gift card for each saliva sample completed. Samples are tested for cotinine, a metabolite of nicotine. Saliva cotinine scores of < 15 ng/ml [43] are considered biochemically confirmed quit. Due to COVID-19 related restrictions, we are unable to collect in-person expired CO at the site for participants who are using NRT or e-cigarettes at the time of their assessment. Consequently, all participants who report nonsmoking at follow-up are sent saliva collection kits and concurrent use of NRT and e-cigarettes is documented at the time of sample collection.

### Participant surveys

Surveys in English or Spanish are administered to patients at baseline, 3-month, and 6-month timepoints. Surveys may be completed electronically via web-based EASEE-PRO platform, or in exceptional cases over the phone with a blinded-to-treatment-arm research assistant or paper by mail. Data is captured and stored securely in the EASEE-PRO system. Patients are mailed a $20 gift card for each survey completed.

#### Secondary smoking outcomes

7-day point-prevalence self-report abstinence is collected at 3- and 6-month follow-up. At baseline, 3-month, and 6-month timepoints, patients are asked: in the past 30 days 1) how many days did they smoke cigarettes, and 2) how many cigarettes per day they typically smoked. ‘Significant reduction’ in smoking is defined as > 50% reduction in cigarettes smoked from baseline to 3- and 6-month follow-ups.

#### Sociodemographic characteristics

Information on sex, age, language, marital status, race, ethnicity, education level, employment status, urban/rural place of residence, zip code, health insurance, and income relative to medical expenses [45] are collected.

#### Smoking history

Number of years a patient has smoked, daily smoking rate, 24-h quit attempts, and other tobacco product use is measured with items adapted from the NCI Cancer Patient Tobacco Use Questionnaire (Q-TUQ) [42, 46]. Nicotine dependence is measured using the Heaviness of Smoking Index from the Fagerstrom Test for Nicotine Dependence (FTND) [47–49].

#### Alcohol use

The Alcohol Use Disorder Identification Test (AUDIT C), [50] a brief 3-item screening test, is used to assess heavy drinking and alcohol dependence.

#### Cancer clinical characteristics

Cancer type, date of diagnosis, stage, and treatment are obtained from sites.

#### Health belief model and self-regulation model measures:

*Emotional distress* is assessed using the Distress Thermometer such that participants are asked to rate their current distress level, on a scale from “0” (No distress) to “10” (Extreme distress) [51, 52].

*Patient coping* is assessed using a 1-item, 11-point scale which evaluates patient ability to cope with stress. Patients are asked to assess and rank how able they are to cope with their current life stressors, ranging from “0” (Not at all able) to “10” (Very much able).

*Anxiety and Depression* are assessed using the PROMIS Item Bank Emotional Distress-Anxiety Short Form 4a, which measures severity of anxiety and depression symptoms over a 7-day period [53, 54]. Patients are asked to respond to a series of statements (e.g., “My worries overwhelmed me” and “I felt hopeless”) with a ranking on the 5-point Likert scale, ranging from “1” (Never) to “5” (Always).

*Cancer stigma* is measured using modified Internalized Stigma and Constrained Disclosure subscales of the Lung Cancer Stigma Inventory (LCSI) [49]. The survey is designed to assess whether patients have experienced stigma since their cancer diagnosis (i.e., “I have blamed myself for having cancer”). Statements are scored using a 5-point Likert scale ranging from “1” (Not at all) to “5” (Extremely). The LCSI has psychometric evidence demonstrating validity and reliability [55, 56].

*Beliefs concerning cessation medications* (e.g., nicotine replacement therapies) are assessed using a modified version of the Attitudes about Nicotine Replacement Therapy Scale (ANRT-12). The ANRT-12 asks about thoughts on using nicotine replacement therapy (e.g., “NRT is easy to use”) using a 5-point agreement scale ranging from “1” (Strongly disagree) to “5” (Strongly agree) [57].

*Perceived benefits of quitting smoking* (e.g., decreasing risk of cancer recurrence, increasing treatment efficacy, etc.) is assessed using a 5-item questionnaire scored on a scale ranging from “0” (Not at all) to “10” (Very much) [58].

*Self-efficacy to quit and the importance of quitting* are assessed using two 1-item, 11-point measurements with a scoring scale ranging from “0” (not confident at all or not important at all) to “10” (very confident or very important) [39].

*Smoking stigma and beliefs concerning the stigmatization of smoking* are assessed using a 6-item agreement scale, ranging from “1” (Strongly disagree) to “5” (Strongly agree), with statements such as “I have avoided telling others that I am a smoker” and “I have worried that others will view me unfavorably because I am a smoker” [59].

*Physical symptoms* of acute nicotine withdrawal (e.g., irritable and poor concentration) within a 24-h period, are assessed using a single item from the Mood and Physical Symptoms Scale (MPSS) [60] scored on a 5-point scale ranging from “1” (Not at all) to “5” (Extremely).

*Environmental influences* (e.g., second-hand smoke exposure and perceived social and provider cessation support) are assessed using 2 questions from the 2008 National Social Climate Survey of Tobacco Control and the PROMIS emotional and informational support 4a short forms [40, 53, 61, 62].

## Measures and data collection, sources, and timeline: implementation outcome measures and data collection

Implementation outcomes are assessed via reports from patients (e.g., participant surveys and exit interviews) and NCORP site staff (e.g., staff surveys and focus groups). Table 2 provides more details on implementation outcomes, data sources and time points for data collection.

**Table 2:** Description of implementation outcomes and measurement for Smokefree Support Study 2.0

Implementation Outcome	Description	Measurement	Data Source	Time Point
Acceptability	Site staff perceived acceptability of tobacco treatment intervention (e.g., met needs, provided cessation assistance wanted, helpfulness and quality of assistance.)	Site staff surveys	NCORP Site Staff	Baseline (before trial enrollment), 12 months, 24–36 months follow-up
	Open-ended questions about aspects of the program most/least helpful, challenges faced to participation, and topic and program recommendations	Site staff focus group interviews	NCORP Site Staff	Following completion of trial enrollment
	Patient satisfaction with content/delivery of their randomly assigned tobacco treatment	Participant 6-month follow up survey	Study participants	Patient 6-month follow-up
	Program engagement (recruitment video, reasons for enrolling), intervention (counseling components and medication), remote delivery and assessments, tobacco counselor, and oncology team support	Post-treatment participant exit interview	VIT participants	Following completion of patient 6-month follow up survey
Organizational Readiness	Site program uptake, Site engagement in implementation facilitation activities	Patient Screening Log Study administrative data	NCORP Site Staff MGB research staff	Weekly Monthly
Appropriateness	Perceived fit and relevance of the VIT and EUC interventions from the perspective of representative site staff	Site staff surveys	NCORP Site Staff	Baseline (before trial enrollment),12 months, 24–36 months follow-up
		Site staff focus group interviews	NCORP Site Staff	Following completion of site participant enrollment
Treatment Fidelity	Delivery of all components of the Smokefree 2.0 Study Treatment Interventions (EUC and VIT)	Tracking of NCI Quitline sessions and NRT dispensed (EUC)	NCI Quitline, EUC participants	Twice yearly
		Collection of data at follow up timepoints regarding whether participants were advised to quit/referred to NCI Quitline by oncology providers	EUC participants	Patient 3-month and 6-month follow -up surveys
		Tracking of number of contacts, session content and completion, NRT dispensed, and any intervention modifications (e.g., counselor adherence to the VIT manual, medication changes) (VIT)	MGB research staff, VIT participants	Weekly
Cost	Incremental cost per quit of the VIT intervention relative to the EUC control over the 6-month follow-up period	Tracking of local staff effort (time x base salary) related to eligibility screening, recorded in the Patient Screening Log	NCORP Site Staff	Weekly
		Counseling delivery costs and NRT delivery costs recorded in MGB database	MGB research staff	Weekly
Feasibility	Ease of delivery and suitability for routine care of the VIT and EUC interventions	Site staff surveys	NCORP Site Staff	Baseline (before trial enrollment),12 months, 24–36 months follow-up
		Site staff focus group interviews	NCORP Site Staff	Following completion of site participant enrollment
Penetration/Reach	Patient participation rate at each site, reasons for study ineligibility and refusal, comparison of sociodemographic and cancer variable characteristics of enrollees and refusers of the study	Patient Screening Log, NCORP Admin data	NCORP Site Staff	Weekly
Sustainability	Resources needed and preference for a site-based centralized tobacco treatment program	Site staff surveys	NCORP Site Staff	Baseline (before trial enrollment),12 months, 24–36 months follow-up
		Site staff focus group interviews	NCORP Site Staff	Following completion of site participant enrollment

### Patient exit surveys and interviews (acceptability)

After completion of their 6-month follow-up surveys, approximately 40 randomly selected VIT participants will offered the opportunity to participate in an audio-recorded remote individual exit interview. Selection is stratified based on reported smoking status (i.e., quit or still smoking) at 6-month follow-up. These interviews follow a semi-structured interview guide and are conducted by study staff remotely. Sample questions include: “What was most helpful about the counseling program?” and “Please tell me about your experience getting the video and camera to work with your tobacco counselor.”

### Staff surveys (intervention feasibility, acceptability, appropriateness)

Prior to beginning patient enrollment at the time of site activation (baseline), the CCDR lead provides information about practice characteristics including safety net designation, minority/underserved NCORP status, geographic location, practice volume, provider mix and ownership type, and tobacco cessation services that are available for patients. Approximately 8–10 oncology clinicians and staff (e.g., site coordinators and support staff) also complete surveys about the implementation of tobacco use assessment and treatment. The feasibility, acceptability and appropriateness of all components of the tobacco treatment interventions are rated on a 6-point ordinal scale [35]. Organizational readiness for implementing tobacco treatment is measured using a modified (i.e., specified “tobacco use assessment and treatment” in place of “this change”) 10-item Organizational Readiness for Implementing Change (ORIC) [36] survey, which assesses two subscales, change commitment and change efficacy, on a 5-point ordinal scale with verbal anchors ranging from ‘agree to disagree’. Baseline site staff surveys are administered prior to the first patient enrollment at their site. Follow-up surveys are conducted 12–15 and 24–36 months following the baseline survey. Site staff are offered (depending on site policy) renumeration of a $20 gift card for completing each survey.

### Staff focus groups (acceptability, feasibility, and sustainability)

A site focus group interview is conducted with representative staff from each participating NCORP site 24–36 months post site activation following the completion of the final staff survey via videoconferencing software. The interviews last approximately 60 min, and site staff participants are remunerated $40 each. The interview assesses implementation processes including barriers and facilitators for sustaining tobacco treatment as routine cancer care practice. The questions for our focus group interviews are guided by the Consolidated Framework for Implementation Research (CFIR) [37] and address 1) the tobacco treatment intervention, 2) inner setting (site characteristics), 3) outer settings (external influences/policies), 4) individuals involved, and 5) the process for sustaining tobacco treatment following trial completion.

#### Cost

We assess NCORP staff time required to screen all patients for smoking status and to collect eligibility data using a weekly Patient Screening Log. All staff time costs are estimated based on national average wages by job type. Counseling delivery costs include the counselors’ time (efforts to contact patients, time delivering counseling, record keeping time and team coordination time) and supervisors’ time (team coordination time), all of which are tracked within study databases. NRT costs (staff time, medications, and shipment) are estimated using national average retail prices. Overall costs are standardized per randomized study participant across sites for the cost-effectiveness.

#### Treatment fidelity

For VIT intervention participants, the number of counseling contacts, session content, and NRT dispensed are documented. For EUC participants, information on the number of Quitline sessions and any NRT dispensed are obtained from Quitline vendors. VIT sessions are recorded. At the conclusion of each counseling session, the TTS completes a checklist of adherence to the treatment protocol. TTS participate in a weekly peer supervision meeting where session audio files are randomly selected for review. The counselors rate each other’s adherence to the counseling protocol as well as adherence to MI principles.

#### Participant reach and site adoptions

Site coordinators document all current smokers identified at each site. Using a weekly site Patient Screening Log, we monitor the number of eligible patients who have been approached and the number of eligible patients who have enrolled. Reasons for ineligibility, refusal, and characteristics of refusers, ineligibles, and dropouts are documented. We also track site uptake and engagement in study activities (i.e., participation in monthly calls and patient program enrollment).

## Methods: data analyses

### Power calculations

Based on our previous trial, [30] we estimate that the 7-day point-prevalence smoking abstinence at 6-month follow-up will be 15% for the EUC group and 32% for the VIT group. The study will have 80% power to detect a 17% difference in 7-day point-prevalence tobacco abstinence with a two-sided significance level of 0.01 with 280 participants. We estimate that 10% of participants will die or be lost to follow-up due to other sources of attrition (e.g., serious illness, loss of interest and preference for a different program) within 6-months of enrollment, so an additional 10% will be recruited (final target *n* = 308).

### Quantitative analyses

We will examine the frequency distributions of all variables. We will compare the baseline characteristics between arms to assess whether randomization distributed covariates evenly. Outcome analyses will follow an intent-to-treat model, and we will initially classify participants who are lost to follow-up and those who do not provide a saliva sample as current smokers. Additionally, we will explore site heterogeneity, covariate distributions, missingness models, and differential dropout in the groups. We will assess the need for probability-of-completion weights to obtain unbiased estimates of treatment effect [63]. We will assess whether data are missing at random. Finally, we will perform sensitivity analyses by using a complete case analysis and by using multiple imputation for missing data [64].

#### Aim 1

We will conduct univariate and multivariable analyses to examine the association between treatment group and smoking outcomes. Chi-square tests will compare the outcomes between treatment groups at each follow-up. Generalized A Estimating Equations (GEE) approach will be used to study the treatment effect over time, incorporating 3- and 6-month follow-up data. This will account for the repeated measures structure of observations within the same individuals over time and allow for analysis of incomplete data across time.

#### Aim 2

An exploratory analysis will assess moderator effects on treatment effectiveness. Of primary interest are patient characteristics: sociodemographic factors, smoking characteristics, cancer, and treatment variables. The effects of NCORP site characteristics (e.g., geographic location and clinic volume) and baseline organizational readiness (a composite average ORIC score will be calculated from each sites’ surveys) will be tested using regression models to determine their association with smoking abstinence.

To explore the impact of intervention targets, we will also conduct linear and logistic regression models with the treatment group as the independent variable, the SRM and HBM targets at follow-up as the dependent variable, and the SRM and HBM target at baseline as a control. We will test for interactions between intervention and these factors to determine whether effects vary among subgroups. We will also conduct exploratory analyses to assess the relationship between changes in HBM and SRM constructs (BL to 3 & 6 months) and treatment group and effectiveness outcomes.

#### Aim 3

We will use descriptive statistics to summarize implementation outcomes (Feasibility, Acceptability, Reach, Fidelity, and Cost) and to conduct treatment group comparisons on relevant treatment implementation outcomes.

##### Feasibility

Staff participants rate perceived feasibility of the tobacco treatment interventions being tested in this trial [35] on a 6-point ordinal scale ranging from ‘Strongly Disagree’ (0) to ‘Strongly Agree’ (5). Aggregate and intervention-specific summary scores for tobacco treatment will be derived.

##### Acceptability

Patient participant satisfaction with various aspects of the tobacco treatment interventions will be evaluated through 6-month participant survey responses and post-treatment exit interviews with a subset of patient participants. Acceptability ratings will be compared between the arms.

##### Reach

We will compare proportion of eligible smokers who participate at each NCORP site using chi-square tests.

##### Fidelity

Using participant self-reported survey and counselor process data, we will evaluate study treatment and non-study treatment (i.e., site-specific usual care and outside resources) utilization. We will dichotomize medication and counseling use into low vs. high (> 8 weeks of NRT; > 8 sessions) levels of treatment utilization [65]. Within each group, we will use chi-square tests and ANOVAs to explore the association between participant characteristics and treatment utilization. We will use chi-square tests to compare the association of treatment utilization (level of medication and counseling use) on smoking outcomes. To predict smoking outcomes, we will use logistic regression models, which will include medication and counseling use levels, adjusting for confounders.

##### Cost

We will calculate the incremental cost per quit of the intervention relative to EUC over the 6-month follow-up period as follows: (total per-person costs of intervention – total per-person costs of EUC)/ (cessation rate with the intervention – cessation rate with EUC) [66]. Cessation rates will be based on the primary outcome findings. Statistical uncertainty in cost and effectiveness inputs will be incorporated into the incremental cost per quit comparisons using Monte Carlo simulation methods allowing us to determine whether these ratios are significantly different from zero and allowing us to assess the proportion of simulation outcomes above or below relevant thresholds. The robustness of the cost-effectiveness ratio estimates will be further examined in sensitivity analyses in which each parameter is varied, singly and in combination, through plausible ranges. We will also generate “best case” and “worst case” analyses. Using Wilcoxon rank sum tests and trend tests (Cochran–Armitage), we will explore patient and site characteristics associated with implementation outcomes.

## Qualitative analyses

Focus group interviews will be recorded, transcribed, and analyzed using NVivo 12 qualitative software [67]. Administrative data (monthly CCDR study meeting minutes, weekly site screening data logs, and emails from sites to study staff) will undergo content analyses by coders, who will conduct an iterative process to develop the framework, categories, and coding plan for each analysis. The focus group interviews will be coded with attention to the CFIR domains [37]. To ensure coding reliability, coding discrepancies will be resolved through discussion. Coding will continue until a high level of reliability (Kappa =  > 0.80) is established. The study PIs will provide review of the analyses.

A convergent, parallel mixed methods design will enhance the program implementation evaluation (methodological triangulation); specifically, quantitative, and qualitative data will be combined to determine the convergence, divergence, and relationships between the survey and qualitative results. We will triangulate data from the different sources (participant survey, exit interview and administrative data) to strengthen the effectiveness and implementation findings.

All study findings will be presented to the EA NCORP Advisory Committee, which includes PIs from participating sites, and at biannual EA Group meetings, to plan for future implementation steps within the EA NCORP network.

## Methods: monitoring

### Risk Assessment

In the unlikely event that a participant is determined to be at potential acute psychiatric risk, study investigators notify the cancer care team of the participant’s risk status and recommend psychiatric evaluation. Additionally, tobacco treatment counselors, trained and supervised by licensed psychologists, routinely assess for suicidality when potential safety issues arise.

### DSMC

The trial is monitored by the EA Data Safety and Monitoring Committee (DSMC) comprised of 9 independent members without direct association to the trial. The committee includes experts in the fields of oncology, radiology, biostatistics, and medical ethics. The DSMC meets biannually to review ongoing patient safety, adverse events, study progress, and data integrity. When appropriate, the DSMC will review interim analyses of outcome data. Only the study statistician and the DSMC members will have access to interim analyses of outcome data.

## Discussion

Continued smoking after cancer diagnosis is common, yet tobacco cessation services are not standard practice in cancer care. Research shows patients with a cancer diagnosis who smoke are not often advised to quit [68, 69], and are not provided cessation services (e.g., counseling and medications) to assist them with quitting and/or maintaining their quit status [70–72] even though tobacco use following a cancer diagnosis contributes to adverse health outcomes, including disease recurrence, development of secondary tumors, and diminished treatment response.

This trial builds upon previously published findings from the Smokefree Support Study 1.0, which demonstrated the effectiveness of an intensive tobacco treatment delivered in-person and via phone among patients recently diagnosed with cancer [30]. To the best of our knowledge, this is the first study to deliver tobacco treatment virtually to recently diagnosed patients treated nationally at community cancer centers. There has been much recent enthusiasm [73] for the use of remote videoconference for tobacco treatment. Videoconferencing improves access to treatment by bringing tobacco counselor expertise directly to patients (synchronous visits) and into community cancer centers. This randomized trial will add to the knowledge on the clinical effectiveness and implementation challenges of a virtual intervention for tobacco treatment in cancer care.

The successful translation of evidence-based tobacco treatment should be informed by implementation science research that documents the process of intervention uptake in cancer care practices. First, this study uses a well-established implementation science framework, the Consolidated Framework for Implementation Research (CFIR) [37], to assess contextual factors influencing intervention uptake within the NCORP. Findings will identify key factors influencing implementation of tobacco use assessment and treatment and determine the best strategies for implementing tobacco cessation for broad national dissemination into community oncology care settings. Second, the trial’s cost analyses will help guide other networks, and organizations in community oncology care, plan for the adoption and delivery of VIT, if successful. Study investigator will communicate trial results in peer-reviewed publications. Datasets will become available in accordance with journal policy.

### Limitations

Despite the innovations of this study, there are several limitations to consider. First, there is substantial variation among participating sites including institutional resources, staffing, workflows for identifying current smokers, and familiarity with NCORP operations. This will be explored in moderator and implementation process analyses. Given the number of and heterogeneity among participating NCORP sites, the standardized collection of data on patients who participate versus those who decline or are ineligible is not feasible. While this is a common limitation across trials conducted through the NCORP network, we acknowledge the limitations associated with the lack of comprehensive data to evaluate potential participation bias. Second, we recognize the potential for varying cancer care treatment pathways (e.g., unanticipated extended hospitalizations) that disrupt tobacco treatment, and therefore, the TTSs flexibility in how they schedule counseling sessions. We will also analyze these variations on outcomes. Third, VIT is a combined counseling and medication treatment, making it difficult to determine the effects of medication or counseling alone. Again, the combined and isolated effect of medication and counseling on outcomes will be explored in data analysis. Finally, this study launched before the emergence of COVID-19 and has been impacted by pandemic-related disruptions to healthcare delivery. This includes significant obstruction and delay to recruitment efforts, sites transferring or furloughing staff, and institutions suspending research activities for extended periods of time. We acknowledge that these obstacles are not exclusive to this study.

## Trial status and modifications

This project was initially funded in February 2018, and we subsequently obtained multiple IRB approvals. We started the site activation processes in May 2019 and closed site enrollment in December 2020. Patient enrollment began in August 2019 and is on-going. We anticipate ending patient enrollment in Fall 2022. We expect to finalize data collection in Spring 2023 and will begin analyzing interim data. The research team has made several modifications to the protocol to facilitate implementation of the trial within the NCORP. These included 1) eliminating severe psychiatric illness ineligibility criteria (due to inability to chart screen), 2) changing the Quitline referral process in EUC from having site staff refer participants to the local state Quitline to a process where participants receive centralized trial-based electronic referral to the NCI Quitline, 3) making viewing of the recruitment video optional, 4) allowing participants to complete some counseling sessions via telephone when needed due to technical issues, 5) adding text outreach for counselors to follow-up with patients, and 6) allowing patients to complete follow-up surveys via mail. Additionally, several protocol changes were related to pandemic-related restrictions, such as allowing site activation on a rolling basis, adjusting sites’ follow-up survey completion period, discontinuing the distribution of CO monitors to sites and mailing all patients who report a quit status the salivary collection kit. All changes were approved by the appropriate Institutional Review Boards.

## Data Availability

Not applicable for this manuscript as it is a protocol paper and there is no data associated with this paper. Future publications with datasets associated with this trial will become available in accordance with journal policy.

## Abbreviations

NCI: National Cancer Institute
NCORP: National Cancer Institute Community Oncology Research Program
VIT: Virtual Intervention Treatment
EUC: Enhanced Usual Control
NRT: Nicotine Replacement Therapy
NCCN: National Comprehensive Cancer Network
TTS: Tobacco Treatment Specialist
CIRD: Centralized Institutional Review Board
MGB: Massachusetts General Brigham
MSKCC: Memorial Sloan Kettering Cancer Center
SRM: Self-Regulation Model
HBM: Health Belief Model
CFIR: Consolidated Framework for Implementation Research
EA: ECOG-ACRIN Cancer Research Group
CCDR: Cancer Care Delivery Research
EASEE-PRO: EA Systems for Easy Entry of Patient Reported Outcomes
MI: Motivational Interviewing
CTTTP: Council for Tobacco Treatment Training Programs
Q-TUQ: NCI Cancer Patient Tobacco Use Questionnaire
FTND: Fagerstrom Test for Nicotine Dependence
AUDIT C: Alcohol Use Disorder Identification Test
LCSI: Lung Cancer Stigma Inventory
ANRT-12: Attitudes about Nicotine Replacement Therapy Scale
MPSS: Mood and Physical Symptoms Scale
ORIC: Organizational Readiness for Implementing Change
DSMC: EA Data Safety and Monitoring Committee
GEE: Generalized Estimating Equations
